# Manipulations of word frequency reveal differences in the processing of morphologically complex and simple words in German

**DOI:** 10.3389/fpsyg.2013.00546

**Published:** 2013-08-22

**Authors:** Maria Bronk, Pienie Zwitserlood, Jens Bölte

**Affiliations:** Institute for Psychology, Westfälische Wilhelms-Universität MünsterMünster, Germany

**Keywords:** lexical decision, morphology, compound processing, word frequency, German

## Abstract

We tested current models of morphological processing in reading with data from four visual lexical decision experiments using German compounds and monomorphemic words. Triplets of two semantically transparent noun-noun compounds and one monomorphemic noun were used in Experiments 1a and 1b. Stimuli within a triplet were matched for full-form frequency. The frequency of the compounds' constituents was varied. The compounds of a triplet shared one constituent, while the frequency of the unshared constituent was either high or low, but always higher than full-form frequency. Reactions were faster to compounds with high-frequency constituents than to compounds with low-frequency constituents, while the latter did not differ from the monomorphemic words. This pattern was not influenced by task difficulty, induced by the type of pseudocompounds used. Pseudocompounds were either created by altering letters of an existing compound (easy pseudocompound, Experiment 1a) or by combining two free morphemes into a non-existing, but morphologically legal, compound (difficult pseudocompound, Experiment 1b). In Experiments 2a and 2b, frequency-matched pairs of semantically opaque noun-noun compounds and simple nouns were tested. In Experiment 2a, with easy pseudocompounds (of the same type as in Experiment 1a), a reaction-time advantage for compounds over monomorphemic words was again observed. This advantage disappeared in Experiment 2b, where difficult pseudocompounds were used. Although a dual-route might account for the data, the findings are best understood in terms of decomposition of low-frequency complex words prior to lexical access, followed by processing costs due to the recombination of morphemes for meaning access. These processing costs vary as a function of intrinsic factors such as semantic transparency, or external factors such as the difficulty of the experimental task.

## Introduction

Is a *doorstep* a *threshold* to our mental lexicon, or do we have to go through the door and a step beyond to access this word? On the path from visual input to meaning, are words such as *tablecloth*, *usurping*, and *premature* parsed into their constituent morphemes, or are they stored as full word forms? If they are parsed, does this happen very early (before lexical access), early (during lexical access), or late (after access to full word-forms)? The question whether morphologically complex words are parsed into their constituents has been of scientific interest for a long time (Taft and Forster, 1975) and has not been unequivocally answered yet (cf. Amenta and Crepaldi, 2012). It is known that many factors influence the processing of morphologically complex words, among which are differences between languages, differences depending on the input modality (visual vs. auditory), on the type of morphological complexity (inflection, derivation, compounding), and on features particular to individual words, such as, for example, the degree of semantic transparency. In the research reported here, we investigated the processing of visually presented compounds. Therefore, in what follows, we focus on data and models for reading complex words. Given our emphasis on the visual processing of compounds, presented without context to adult native speakers, we largely restrict our discussion of existing data to these issues.

An important question is whether the processing of all complex words follows the same route, or whether there are different options depending, for example, on frequency of use, or the degree of semantic transparency. Almost four decades ago, Taft and Forster (1975) used verbs in a visual lexical decision task to show that prefixes are stripped-off from their stems prior to lexical access. Since then, psycholinguistic research has seen the birth of various models and numerous scientific publications supporting each of them. Taft developed a model (cf. Taft, 2004; Taft and Ardasinski, 2006; Taft and Nguyen-Hoan, 2010) claiming morphological decomposition prior to mental lexicon access for any morphologically complex word, whereas Butterworth (1983) proposed a completely opposite hypothesis, claiming that all known words are stored as full forms in the mental lexicon. Between these extreme positions, other models have emerged. One is the supralexical model of Giraudo and Grainger (2000), which states that all words are retrieved as full forms first, and morphological features are accessible only afterwards. Finally, dual-route approaches such as the Morphological Race Model (MRM) by Schreuder and Baayen (1995), or the Augmented Addressed Morphology Model (AAM) by Caramazza et al. (1988), assume that both full-form access and access via decomposed constituents is possible. In the MRM, the route taken depends on factors such as the frequency of the full word form and its constituents, or semantic transparency. The AAM assumes that lexical access is possible via full-forms and via morphological decomposition, with full-form access being the normal (and faster) route for known words, whereas the constituent-based access is faster only for previously not encountered words.

Morphological effects have been studied with numerous designs, using derived words (e.g., Longtin and Meunier, 2005; Kuperman et al., 2010), inflected words (e.g., Lehtonen et al., 2007; Leinonen et al., 2009), and compounds (e.g., Fiorentino and Poeppel, 2007; Ji et al., 2011; Juhasz and Berkowitz, 2011). What the diversity in models and data shows, is that many questions about morphological complexity are still unresolved, and we hope to shed light on some of them. Here, we concentrate on (visually presented) German compounds, addressing the following questions: Are there morphological effects at the word-form level in reading complex German words? Next, if decomposition takes place—does it come at some processing costs, and if so, what factors influence these processing costs? There are data from several languages with respect to the first issue. Rastle and colleagues (Rastle et al., 2004; Rastle and Davis, 2008) found significant priming effects in English, with masked visual priming, when the prime was derived from the target (*teacher—teach)*, but also when the relationship was not morphological but rather accidental *(e.g*., *corner—corn*). This provides clear evidence for early morphological decomposition of derived words—even of pseudoderived words—, which has been replicated in other languages (for French: Longtin and Meunier, 2005; for Russian: Kazanina et al., 2008).

With respect to compound reading, there is support for morphological decomposition from several languages. Fiorentino and Poeppel (2007) report evidence for morphological decomposition prior to word-form access. Their method features the comparison of morphologically complex words with monomorphemic words, matched with respect to length, frequency, and other linguistic factors. They compared English compounds and simple words, using visual lexical decision with simultaneous MEG registration. Compounds were recognized significantly faster than frequency- and length-matched monomorphemic nouns, and the MEG signal also revealed evidence for early decomposition. Crepaldi et al. (2013) reported that the morphemes *moon* and *honey* from transposed-constituent pseudocompounds (^*^moonhoney), activate the representation of *honeymoon.* Lemhöfer et al. (2011) showed that orthotactic cues at the morpheme boundaries of Dutch compounds led to faster responses compared to compounds lacking such cues, thus providing evidence for morphemic parsing. But not all evidence speaks in favor of decomposition, and there are noticeable differences between languages. In a review of data on Mandarin Chinese, Dronjic (2011) concluded that full-form access predominates Chinese compound processing, although recent findings indicate some flexibility (Cui et al., 2013). Bertram and Hyönä (2003) obtained evidence for decomposition for long Finnish compounds, but not consistently for short ones. Hyönä (2012) concludes that short compounds that can be viewed within one fixation are processed along a whole-word route. Longer compounds, especially those with hyphenated morpheme boundaries, encourage decomposition. Finally, and closely related to our own study, Ji et al. (2011) reported processing costs for morphologically complex words, but not in all circumstances. They found shorter lexical-decision times for semantically transparent English compounds than for matched monomorphemic nouns. This advantage disappeared and even turned into a disadvantage for semantically opaque compounds, when the experimental design encouraged decomposition. Ji et al. explain their findings in terms of early morphological decomposition, followed by necessary constituent integration, to gain access the word's meaning. Depending on various factors (semantic opacity, experimental manipulations), this integration may outweigh the processing advantages due to decomposition, and therefore compound processing may take longer than the processing of a morphologically simpler word.

So, there is evidence in favor of early decomposition (Fiorentino and Poeppel, 2007; Rastle and Davis, 2008), for processing costs later on, and for differences as a function of semantic transparency (Ji et al., 2011). But as often is the case, most of the evidence comes from English, and data from other languages (Finnish, for example) show a different pattern. It is thus important to provide further data, from different languages, to enlighten these issues.

To explore whether early decomposition takes place during the processing of morphologically complex words of German, we used German noun-noun compounds and matched them in length and surface frequency with monomorphemic nouns. We ensured that the constituents of the compounds were always of higher frequency than the full compounds. As has been known for a long time, the frequency of occurrence of a word determines the speed with which it is recognized (cf. Andrews, 1986). The idea is that word frequency is a feature of word forms. Frequent word forms are either accessed before infrequent ones (Forster, 1976), or have higher resting levels of activation (McClelland and Rumelhart, 1981). Frequency can thus be used, and has been used, as a diagnostic tool to address issues of full-form storage and decomposition (e.g., Alegre and Gordon, 1999; Baayen et al., 2010). If morphologically complex words are treated in the same manner as morphologically simple words during word recognition, they should be recognized with a similar latency as monomorphemic words, when matched in overall frequency and length. If, however, morphological decomposition takes place upon lexical access, compounds should be recognized faster than monomorphemic words, because of their more frequent constituents.

There is ample evidence that the frequency of compound constituents play a role during visual word recognition, for English (cf. Juhasz et al., 2003; Andrews et al., 2004; Wang et al., 2010), Spanish and Basque (Duñabeitia et al., 2007, 2008), Dutch (Kuperman et al., 2009), and Finnish (Hyönä and Pollatsek, 1998; Pollatsek et al., 2000). Effects of constituent frequency are interpreted in favor of decomposition, or for the existence of two routes to visual-word recognition. However, evidence for German remains scarce (cf. Böhl, 2007).

If decomposition takes place, the constituents have to be re-assembled at some point, to distinguish existing compounds, such as *doorstep*, from ones that do not exist, such as *doorwater*. This re-assembly may come at the price of extra processing costs, which then might consume any head-start advantage. Thus, given the task and timing, compounds might be recognized as slowly as, or even more slowly than, monomorphemic words due to these re-assembly processes (cf. Taft and Ardasinski, 2006; Ji et al., 2011). The most prominent reason for re-assembly applies inside and outside of the laboratory, and concerns the fact that the meaning of any compound is not the mere sum of the meanings of its constituents. To integrate the meaning of the constituents, their relational structure needs to be retrieved (cf. Gagné and Spalding, 2009). Even for semantically transparent compounds, the relation between modifier and head can vary considerably, as in *cheesecake*, *cupcake*, and *wedding cake.* This is even more relevant for compounds that are semantically opaque, for which the relationship between the meaning of the compound and the meaning of its constituents is opaque or absent (e.g., *soap opera; hogwash*). As a consequence, some models assume that semantically opaque words, though morphologically complex, are accessed more quickly via their whole-word forms (Schreuder and Baayen, 1995).

Although the results are somewhat mixed (cf. Libben, 1998), there is evidence that (partially) opaque compounds are decomposed, for English (Libben et al., 2003; Frisson et al., 2008), Greek, Polish, French, and Bulgarian (Jarema et al., 1999; Kehayia et al., 1999), Dutch (Zwitserlood, 1994), and Finnish (Pollatsek and Hyönä, 2005). Semantic transparency does have an impact on gaze durations in reading English compounds (Juhasz, 2007) and fully opaque compounds seem to be treated as monomorphemic words (Zwitserlood, 1994; Libben, 1998). We therefore also manipulated the semantic transparency of compounds in separate experiments, to further investigate the influence of semantic transparency on word recognition in compound reading.

In order to assess potential processing benefits and costs, in Experiment 1 we used pairs of semantically transparent compounds with different constituent frequencies, matched in overall frequency and length to monomorphemic words. When decomposition takes places upon lexical access, a larger head start is expected for compounds with high-frequent constituents than for compounds with low-frequent constituents. A lexical decision task with different levels of difficulty was used, to tax potential differences in processing costs. In Experiment 1a, the pseudowords for the lexical decision task were relatively easy to detect: whether monomorphemic (*instrament*) or complex (*doorstip*, *toirstep*), they clearly diverged from existing word forms. Following Taft (Taft, 2004; Taft and Ardasinski, 2006), we expected word decisions to be fast and easy. Frequent words, even complex ones, might be recognized via full-form access in the context of such easy pseudowords (cf. Taft and Ardasinski, 2006). Given the low overall frequency of the compounds, and the much higher frequency of their constituents, we expected access via decomposition even when pseudowords were easy. We thus hoped to tap into a lexical-access advantage due to constituent frequency. An observation of such an advantage would provide evidence for decomposition. Thus, our design should enable us to distinguish between models that feature early decomposition (e.g., Taft and Nguyen-Hoan, 2010), and those that do not (Butterworth, 1983; Giraudo and Grainger, 2001). In Experiment 1b, the pseudocompounds consisted of combinations of two free morphemes that do not make up an existing compound (*pianocup*, *dressfork*). To distinguish between existing compounds and pseudocompounds, either lookup of the full form is attempted—albeit without success—or the pseudowords are decomposed, and information is needed from the re-assembly or integration stage, to decide whether the combination of two existing morphemes also exists. But even if constituent integration partially devours the potential advantage relative to matched monomorphemic words, there should still be a difference between compounds with high- or low-frequency constituents.

In Experiments 2a and 2b, we used semantically intransparent compounds. The models mentioned above differ in their assumptions concerning semantic transparency. The MRM (Schreuder and Baayen, 1995) assumes that semantically opaque words are processed along a direct route, by the retrieval of their full forms stored in the mental lexicon. This is contrary to Taft's (e.g., Taft and Nguyen-Hoan, 2010) model, which states that all morphologically complex words are parsed, independent of their semantic transparency. To put these assumptions to test, we used transparent compounds in Experiment 1, and opaque compounds in Experiment 2. If semantically opaque words are retrieved from the mental lexicon as full forms, reaction times (RTs) should not differ between compounds and matched monomorphemic words. If, however, opaque compounds are decomposed into their constituents, we should find faster RTs for compounds, because of the higher constituent frequencies. As in Experiment 1, we used different types of pseudowords in Experiments 2a and 2b, to potentially tax the integration stage—if opaque compounds are indeed decomposed. Given the findings of Ji et al. (2011), we might observe different processing costs for semantically transparent and opaque compounds. A joint interpretation of the results of Experiments 1 and 2 should enable us to shed more light on the routes that visually presented morphologically complex words follow as they are processed.

## Experiment 1a

### Method

#### Participants

The experiment was conducted with 31 native speakers of German (mean age = 21 years, 27 females) from the Westfälische Wilhelms-Universität Münster who received course credit or money for their participation. All had normal or corrected-to-normal vision. The local ethics committee approved of all procedures reported for this and the following experiments. In every experiment reported here informed consent was obtained from all participants.

#### Materials

Seventy-two triplets consisting of two German bi-morphemic, semantically transparent noun-noun compounds and one monomorphemic noun, for example, *Papierhut* (paper hat), *Zauberhut* (magic hat), *Margerite* (marguerite), were used. See Table 1 for examples of each stimulus class and an overview of stimulus properties. Appendix lists the complete stimulus set.

**Table 1:** **Experiments 1a and 1b: Mean word frequency and mean word length in number of letters (SD)**.

**Stimulus class**	**Example**	**Experiment 1a**		**Experiment 1b**	
		**Mean frequency (SD)**	**Mean word length (SD)**	**Mean frequency (SD)**	**Mean word length (SD)**
Compounds “high”	*Papierhut* (paper hat)	17.21 (1.81)	8.49 (0.86)	17.21 (1.81)	8.49 (0.86)
Constituent “high”	*Papier* (paper)	9.07 (1.91)		9.07 (1.91)	
Compounds “low”	*Zauberhut* (magic hat)	17.29 (1.82)	8.49 (0.87)	17.29 (1.82)	8.49 (0.87)
Constituent “low”	*Zauber* (magic)	13.58 (1.94)		13.58 (1.94)	
Simple words	*Margerite* (marguerite)	17.29 (1.70)	8.49 (1.04)	17.19 (1.77)	8.47 (0.95)
Simple words (Filler)	*Mikrophon* (microphone)		8.40 (0.90)		8.49 (0.89)
Simple words (Pseudo)	^*^Instrumunt (^*^instrumunt)		8.27 (1.61)		8.49 (0.89)
Compounds (Pseudo “easy”)	^*^Blamentepf (^*^flewer pat)		8.47 (0.87)		–
Compounds (Pseudo “difficult”)	^*^Pianotasse (^*^piano cup)	–	–		8.60 (0.99)

The frequencies of the constituents reported here and hereafter always concern the frequency of these nouns as they occur in isolation, not as a part of any combination. The surface frequency of compounds and simple words within each triplet was matched using the Leipziger Wortschatz–Lexikon (March 2009). In the Leipziger Wortschatz frequency classes can be obtained for each word relative to the frequency of the masculine definite article “*der*,” which is the most frequent word in German. High values code low frequencies, contrary to common usage. In the following, we will use “high” and “low” frequency as is common, even though the relevant class information is numerically opposite.

Surface frequency of the triplets ranged from class 14 to 21 (mean frequency class = 17.26, *SD* = 1.77). Members of a triplet did not differ from each other in more than one frequency class. The compounds of a triplet shared one constituent (*–hut*/*hat* in the example mentioned above) either in modifier (50% of the set) or in head position (50% of the set; German compounds are right-headed). Compounds were selected such that the non-shared constituents, such as *Papier* and *Zauber* in the example, varied in frequency class. This resulted in a “high” frequency set (constituent mean = 9.07, *SD* = 0.23) and a “low” frequency set (constituent mean = 13.58, *SD* = 0.229). Note that the constituents were always more frequent than the compounds (compound mean = 17.26, *SD* = 1.768). The shared constituent had a mean frequency class of 10.06, *SD* = 2.31. Simple words had a mean frequency class of 17.29, *SD* = 0.20. Compounds were thus closely matched with simple words in surface frequency, as well as in word length. Word length ranged from 6 to 11 characters (mean = 8.49, *SD* = 0.092). Triplet members differed in word length maximally by one letter.

Although matching was done on the basis of the Leipziger Wortschatz, the best database available at that time, we checked these frequencies in the new *dlex* database (Heister et al., 2011), which has a more common “words per million”-count. For those items that were also present in dlex, (July 2011) the statistics are as follows: The high-frequency compounds had a mean surface frequency of 43 (*SD* = 0.54; range = 0.008–2.23). The mean frequency of the non-shared constituent was 73.4 (*SD* = 119.56). The low-frequency compounds had a mean surface frequency of.43 (*SD* = 0.75; range = 0.008–4.18). The mean frequency of the non-shared constituent was 6.38 (*SD* = 8.94). The mean frequency of the shared constituent was 51.14 (*SD* = 64.13). The simple words had a slightly higher mean surface frequency of 1.03 (*SD* = 1.51, range = 0.025–8.94).

Seventy-two simple words were added as fillers to the stimulus set, to balance the ratio between compounds and simple words. In addition, 288 pronounceable pseudowords were created by changing one or two vowels or consonants of existing words. There were 144 simple pseudowords (e.g., ^*^Instrumunt), 48 word/pseudoword compounds (e.g., ^*^Weupennest), 48 pseudoword/word compounds (e.g., ^*^Senfsime), and 48 pseudoword/pseudoword compounds (e.g., ^*^Blamentepf). Word length was matched between words and pseudowords. The 72 triplets were evenly distributed across two lists, with the triplets' compounds on different lists. Filler words and pseudowords were evenly distributed. Every participant saw both lists, with eight practice trials placed at the beginning of each. List presentation order was balanced across participants.

#### Procedure

The participants were tested individually in a quiet room, sitting in front of a 17″ computer screen (CTX 1785 XE). They were instructed to decide as quickly and accurately as possible via button press on the keyboard whether the visually presented stimulus was a word or pseudoword. The stimuli were presented in random order, in black 28pt Verdana font on a white background. In all experiments, the visual angle on the stimuli was.8° vertically, and ranged from 2.8 to 6.6° horizontally (6–12 characters width). Viewing distance was 75 cm. A fixation cross initiated the trial and was present for 550 ms. A blank screen, following the fixation cross and present for 500 ms, preceded stimulus presentation, which lasted 1000 ms. RT was measured from stimulus onset for maximally 2500 ms. The inter-trial interval (ITI) was set to 650 ms. The NESU system (New Experimental Set Up, Baumann et al., 1992) was used for stimulus presentation and reaction-time measurement. A recording session lasted about 30 min.

#### Results

Two participants and 9 triplets were discarded from further analyses due to high error rates (more than 20% errors for participants, more than 40% errors in a condition). Because the frequency- and length-matching was done triplet-wise, the stimulus properties are not affected by the triplet-wise exclusion of stimuli. The matching holds, even if many triplets are excluded. After exclusion, the total error rate was 7.1%. See Table 2 for mean RTs and error rates of the different stimulus classes.

**Table 2:** **Experiments 1a and 1b: Errors in percentages and mean RT in ms (SD)**.

**Stimulus class**	**Example**	**Experiment 1a**		**Experiment 1b**	
		**% Error**	**Mean RT (SD)**	**% Error**	**Mean RT (SD)**
Compounds “high”	*Papierhut* (paper hat)	8.4	604 (107)	17.1	685 (101)
Compounds “low”	*Zauberhut* (magic hat)	11.5	633 (119)	19.7	718 (121)
Simple words	*Margerite* (marguerite)	12.4	634 (96)	22.0	721 (107)
Simple words (Filler)	*Mikrophon* (microphone)	4.0	574 (82)	5.5	649 (81)
Simple words (Pseudo)	^*^Instrumunt (^*^instrumunt)	6.4	694 (130)	6.6	784 (195)
Compounds (Pseudo “easy”)	^*^Blamentepf (^*^flewer pat)	3.9	702 (151)	–	–
Compounds (Pseudo “difficult”)	^*^Pianotasse (^*^piano cup)	–	–	13.2	856 (217)

For the analysis of the RT data, trimmed means (5%) per condition averaged over participants (*F*_1_) and items (*F*_2_) served as dependent variable. First, we determined whether Frequency (high, low) and Position of Shared Constituent (modifier, head) interacted with each other. Neither the *F*_1_ (Two-Way repeated measures ANOVA) nor the *F*_2_ (Two-Way ANOVA) showed a significant interaction, all *Fs* < 1. Therefore, the factor Position of Shared Constituent was dropped from further analyses.

A One-Way repeated measures ANOVA (*F*_1_) and One-Way ANOVA (*F*_2_) using the factor Word Type (compounds with high-frequency constituent, compounds with low-frequency constituent, simple words) yielded a significant main effect [*F*_1(2, 56)_ = 21.439, *p* < 0.001, *GG* = 0.864, *partial* η^2^ = 0.434; *F*_2(2, 186)_ = 7.228, *p* = 0.001, *partial* η^2^ = 0.072]. Subsequently, planned *t*-tests revealed that participants responded significantly faster (30 ms) to compounds with a high-frequency constituent than to morphologically simple words: *t*_1(28)_ = 5.772, *p* < 0.001 two-tailed, *d* = 0.30; *t*_2(62)_ = 3.740, *p* < 0.001 two-tailed, *d* = 0.65. They also responded significantly faster (29 ms) to compounds with a high-frequency constituent than to compounds with a low-frequency constituent: *t*_1(28)_ = 6.883, *p* < 0.001 two-tailed, *d* = 0.26; *t*_2(62)_ = 4.184, *p* < 0.001 two-tailed, *d* = 0.61. The mean RT to compounds with a low-frequency constituent did not differ significantly from the RT of morphologically simple words: all *t*s < 1.

#### Discussion

So far, our data give evidence for morphological parsing. Latencies for compounds with a high-frequency constituent were shorter than for compounds with a low-frequency constituent and for monomorphemic words, although surface frequency was matched. This advantage due to constituent frequency can only be explained by access to the constituents, and thus by decomposition. RTs were almost identical to monomorphemic words and to compounds with constituents of lower frequency. This pattern would fit well with full-form access for those compounds. But note that the constituents of the low-frequency compounds were still far more frequent than the matched overall frequency of the compound and the simple word. The overall very low frequency of the compound, relative to the frequency of its constituents, should invite decomposition. But if decomposition and reassembly had come without costs, we would have expected faster responses even for the low-frequency compounds, compared to the monomorphemic words. To further investigate this, in the next experiment the task for the participants was more difficult to accomplish. This was achieved by using pseudowords made up from existing constituents. These can be distinguished from existing words by lookup—checking the lexicon for the existence of a full form—or by checking the existence of the combination, after their constituents have been recognized. Taft and colleagues have shown that the inclusion of such pseudowords clearly invites decomposition (Taft, 2004; Taft and Ardasinski, 2006). This would clearly tax the re-assembly stage more heavily than necessary in the context of pseudowords with nonce stems. As a consequence, the advantage over monomorphemic words due to constituent frequency might be annihilated.

## Experiment 1b

### Method

#### Participants

Thirty-three students (mean age = 21 years, 31 females) were recruited from the Westfälische Wilhelms-Universität Münster. They received course credit or money for their participation. All were native speakers of German and had normal or corrected-to-normal vision.

#### Materials

We replaced 13 simple words in the 72 triplets described in Experiment 1a to match frequency and word length even better, and to replace error-prone words. As before, only semantically transparent compounds were used. See Table 1 for examples of each stimulus class and an overview of stimulus properties, and Appendix for a list of the whole stimulus set. The same criteria (word frequency class and word length in letters) as before were applied.

Seventy-two simple words were added to balance the ratio of compounds and simple words. One hundred and forty-four simple pseudowords were created by replacing one consonant or vowel in an existing word (e.g., ^*^Flunser). Another 144 pseudocompounds were created by combining simple words into a pseudocompound (e.g., ^*^Pianotasse = ^*^piano cup). Word length was balanced between the different classes of stimuli. The stimuli were distributed over two lists, as in Experiment 1a. Each participant saw both lists, with eight practice trials at the beginning of each list.

#### Procedure

The stimuli were presented in random order in black 28pt Verdana font on a white background. A fixation cross initiated the trial and was present for 320 ms. A blank screen, following the fixation cross and present for 360 ms, preceded stimulus presentation, which lasted 800 ms. RT was measured from stimulus onset for maximally 3000 ms. We anticipated that the pseudocompounds consisting of two existing words would make the lexical decision more difficult than the pseudocompounds from Experiment 1a. Therefore, time-out was set to 3000 ms. In this and all following experiments, presentation times were shorter than in Experiment 1a. This was done to keep the testing sessions as short as possible, and to avoid fatigue or boredom on the participants' side. Inquisit software (Inquisit 3.0.4.0, Millisecond Software, 2010) was used for presentation and RT-measurement.

#### Results

The total error rate was 13.0%. See Table 2 for mean RT and error rates of the different stimulus classes. We used the same exclusion criteria as in Experiment 1a for discarding participants and items. The data of 32 participants and 46 triplets were retained in subsequent analyses. Again, because the frequency- and length-matching was done triplet-wise, triplet-wise exclusion does not affect stimulus-properties. As before, trimmed means (5%) per condition, participant, and item served as dependent variable.

Again, RT to compounds did not show a significant interaction of Frequency (compounds with a high-frequency constituent, compounds with a low-frequency constituent) and Position of Shared Constituent (modifier, head): all *Fs* < 1. Therefore, we collapsed the data over the factor Position of Shared Constituent.

A significant main effect for Word Type (compounds with a high-frequency constituent, compounds with a low-frequency constituent, simple words) was found for *F*_1(2, 62)_ = 13.175, *p* < 0.001, *GG* = 0.930, *partial* η^2^ = 0.298 as well as for *F*_2(2, 135)_ = 5.709, *p* = 0.004, *partial* η^2^ = 0.078. Although participants responded about 80 ms slower than participants in Experiment 1a, the response pattern was similar in both experiments. Planned *t*-tests revealed that responses were significantly faster (36 ms) to compounds with a high-frequency constituent than to morphologically simple words: *t*_1(31)_ = 4.120, *p* < 0.001 two-tailed, *d* = 0.34; *t*_2(45)_ = 3.252, *p* = 0.002 two-tailed, *d* = 0.68. Participants also responded significantly faster (33 ms) to compounds with a high-frequency constituent than to compounds with a low-frequency constituent: *t*_1(31)_ = 4.787, *p* < 0.001 two-tailed, *d* = 0.30; *t*_2(45)_ = 3.592, *p* < 0.001 two-tailed, *d* = 0.62. The mean RT to compounds with a low-frequency constituent did not differ significantly from the RT to morphologically simple words: all *ts* < 1, replicating the data pattern of Experiment 1a. In a combined analysis of the data from Experiments 1a and 1b no interaction emerged with the factor “Experiment” (a, b): all *Fs* < 1.

#### Discussion

What the data of Experiments 1a and 1b show is that the type of pseudoword does not differentially affect the processing of the compound stimuli, apart from overall longer RTs, which can be attributed to the differences in general task difficulty. This is evident from the fact that the presence of difficult pseudowords, the non-existing combinations of existing morphemes (e.g., ^*^pianocup), also slowed reactions to monomorphemic words. In fact, the slowing, comparing mean RTs from Experiments 1a and 1b, is very similar for high-frequency compounds (81 ms), low-frequency compounds (85 ms), and monomorphemic words (87 ms). So, the pseudoword manipulation worked, as is also evident from the error rate for difficult pseudowords, which is much higher than that for the easy ones used in Experiment 1a.

Results so far clearly speak in favor of decomposition, even when the pseudowords are easily rejected because of the non-word status of (one of) the morphemes (Experiment 1a). It thus seems that decomposition is rather automatic, at least independent of the processes required for reaching a correct decision on the pseudowords. Integration of constituents does take time, and it seems to consume the constituent-frequency advantage expected for the low-frequency words. But it does not take more time when existing compounds and pseudocompounds are harder to distinguish.

So far, we can only speak to the processing of semantically transparent stimuli, and it remains an open question whether the morphological parsing observed for transparent compounds also applies to semantically opaque compound nouns. We therefore tested such stimuli in two additional experiments with similar manipulations as Experiments 1a and 1b. Given that opaque compound pairs sharing a morpheme are virtually impossible to find, instead of triplets, we used pairs of opaque compounds and monomorphemic words, matched for surface frequency and length. Both constituents were clearly higher in frequency than the full compound.

In Experiments 1a and 1b a high error rate, especially for monomorphemic nouns, could be observed. Since most stimuli are of a low surface frequency and thus appear but rarely in common every-day language, participants seem to be guessing when they are not sure whether the stimulus was an existing word. In the following experiments, we therefore used a questionnaire, which helped us to distinguish between words known to every participant and those that were unknown. Another change involved the use of the new *dlex* data base, instead of the Leipziger Wortschatz, for obtaining frequency counts. This change was motivated by the good results the *dlex* data base obtained in a comparison of different data bases (Brysbaert et al., 2011).

## Experiment 2a

### Method

#### Participants

Thirty-three students from the same population as before (26 females, mean age = 26 years) participated in this experiment. All subjects had normal or corrected-to-normal vision and were German native speakers. They received course credit or € 3.00 for participation.

#### Materials

Experiment 2a made use of pairs of compounds and matched monomorphemic words. Thirty-six semantically opaque bi-morphemic noun-noun compounds (e.g., *Seifenoper*/*soap opera*) were drawn from *dlex* data base (Heister et al., 2011). See Table 3 for examples of each stimulus class, and an overview of stimulus properties. Appendix lists the complete stimulus set.

**Table 3:** **Experiments 2a and 2b: Mean word frequency and mean word length in number of letters (SD)**.

**Stimulus class**	**Example**	**Experiment 2a**		**Experiment 2b**	
		**Mean frequency (SD**	**Mean word length (SD)**	**Mean frequency (SD)**	**Mean word length (SD)**
Compounds “opaque”	*Seifenoper* (soap opera)	0.46 (0.77)	9.33 (1.29)	0.46 (0.77)	9.33 (1.29)
1st Constituent	*Seife* (soap)	42.01 (60.87)		42.01 (60.87)	
2nd Constituent	*Oper* (opera)	47.54 (59.59)		47.54 (59.59)	
Simple words	*Frikadelle* (rissole)	0.45 (0.65)	9.17 (1.11)	0.45 (0.65)	9.17 (1.11)
Simple words (Pseudo)	^*^Nachtigalf (^*^nightingalf)		9.28 (1.23)		9.28 (1.23)
Compounds (Pseudo “easy”)	^*^Leubfrasch (^*^trea frig)		9.33 (1.29)	–	–
Compounds (Pseudo “difficult”)	^*^Schneemusik (^*^snow music)	–	–		9.33 (1.29)

Subsequent frequencies are reported as normalized type frequency (in words per million). The compounds were matched in surface frequency with morphologically simple words, such as *Frikadelle* (*rissole)*. This matching was done item by item. The mean surface frequency of compounds was 0.46, *SD* = 0.76, and the mean surface frequency of the matched simple words was 0.45, *SD* = 0.65. Constituent frequency was always much higher than surface frequency (modifier-constituent mean frequency: 42.0, *SD* = 60.9, head-constituent mean frequency: 47.5, *SD* = 59.6). In addition, compounds and monomorphemic words were exactly matched for word length (mean word length = 9.25, *SD* = 1.2), ranging from 6 to 13 characters.

The compound's level of opaqueness was assessed in a pre-test. Twenty hundred and eight potential semantically opaque bi-morphemic compounds were distributed over two lists. Twenty-six semantically transparent compounds were added to each list, to ensure that participants used the full range of the rating scale. Each list was rated by 51 participants who received € 3.00 for their rating. Participants were asked to determine how well the meaning of a compound could be derived from its constituents by using a six-point scale (1 = “very badly” to 6 = “very well”). There was also an option to mark the word as unknown to the rater. A stimulus was selected for the experiment if it matched all of the following criteria: compound known by at least 80% of the participants, constituent frequency larger than compound frequency, noun-noun compound, possibility to match the compound with a simple word in word length and word frequency. The most opaque compounds that matched these criteria were selected for the experiment. The 36 compounds that entered the experiment had a mean value of 2.42 (*SD* = 0.80) in the opacity-pre-test.

As in Experiment 1a, an equal number of pseudowords were created by replacing one consonant or vowel in an existing word. There were 36 simple pseudowords (e.g., ^*^Klarineste). In addition, we created 36 pseudocompounds. In 12 of them the first constituent of an existing compound was altered by replacing a vowel or consonant (e.g., ^*^Spaubkorn). In another 12 pseudocompounds the second constituent was altered (e.g., ^*^Strandnorb), and in the last 12 pseudocompounds both constituents were altered (e.g., ^*^Leubfrasch). Word length was balanced between the different classes of stimuli. The stimuli were distributed evenly over two lists, along with four practice trials at the beginning of each list. Each participant saw both lists. List presentation order was balanced across participants.

#### Procedure

Participants were tested individually in a quiet room, seated in front of a 17″ computer screen (CTX 1785 XE). They were instructed to judge as quickly and accurately as possible whether the stimuli were words or pseudowords. The participants entered their answers via button press (green and red button for “yes” and “no”) on a Cedrus Response Pad (Cedrus RB-830). All stimuli were presented in random order in black 28pt Verdana font on a white background. Each trial began with the presentation of a fixation-cross (320 ms duration). The target, presented for 800 ms, followed 360 ms after the fixation cross offset. RT was measured from target onset, and time out was set to 2500 ms. Each ITI lasted for 1540 ms. Inquisit software (Inquisit 3.0.4.0, Millisecond Software, 2010) was used for presentation. After the experiment each participant was asked to fill in a questionnaire, in which they had to indicate whether they knew the word stimuli or not. A session lasted about 15 min.

#### Results

After discarding all trials containing words marked as “unknown” by the participants (41 compounds and 178 simple words out of 2376 existing words), the total error rate was 8.4%. See Table 4 for mean RTs and error rates of the different stimulus classes. Due to high error rates (above 20% for participants, above 30% in one condition for item pairs), 4 participants and 7 item pairs had to be excluded from further analysis. Stimuli were excluded pairwise, because the frequency- and length-matching was done pairwise. The same trimming procedure as in Experiment 1 was applied. Latencies were averaged over participants (*t*_1_) and items (*t*_2_). Given our two-condition design, the remaining analyses were conducted with planned *t*-tests. These tests revealed that participants responded significantly faster (57 ms) to compounds than to morphologically simple words: *t*_1(29)_ = 6.239, *p* < 0.001 two-tailed, *d* = 0.38; *t*_2(29)_ = 3.398, *p* = 0.002 two-tailed, *d* = 0.85.

**Table 4:** **Experiments 2a and 2b: Errors in percentages and mean RT in ms (SD)**.

**Stimulus class**	**Example**	**Experiment 2a**		**Experiment 2b**	
		**% Error**	**Mean RT (SD)**	**% Error**	**Mean RT (SD)**
Compounds “opaque”	*Seifenoper* (soap opera)	5.9	646 (138)	6.0	807 (266)
Simple words	*Frikadelle* (rissole)	14.7	703 (163)	13.3	811 (209)
Simple words (Pseudo)	^*^Nachtigalf (^*^nightingalf)	6.9	779 (203)	5.4	902 (269)
Compounds (Pseudo “easy”)	^*^Leubfrasch (^*^trea frig)	7.0	795 (204)	–	–
Compounds (Pseudo “difficult”)	^*^Schneemusik (^*^snow music)	–	–	11.1	963 (271)

#### Discussion

As in Experiment 1a, RTs were significantly shorter for compounds than for matched monomorphemic words. If anything, the effect was numerically larger for opaque than for transparent compounds (57 vs. 29 ms), possibly due to larger frequency differences between surface forms and constituents (although the different frequency measures hamper a direct comparison). This again provides clear support for decomposition, and for a similar processing of semantically transparent and opaque words. Note that we obtain clear evidence for decomposition with easy pseudowords. Even for easy pseudowords Taft and colleagues (Taft, 2004; Taft and Ardasinski, 2006) would have predicted that the RT advantage for compounds over simple words might be cancelled by high processing costs at the recombination stage. Taft and colleagues also emphasize that the recombination of constituents into a unifying representation is necessary for any complex word that cannot be understood by means of access to the meaning of its constituents. This is clearly the case for opaque compounds, and the unifying representations are labeled “lemma.” Semantic information can only be accessed via this lemma, and this extra step demands processing costs. Apparently, in an environment of easy pseudocompounds, this does not consume the advantage due to constituent frequency, and hardly slows down the recognition of compounds, resulting in shorter RTs for opaque compounds than for monomorphemic words.

In Experiment 2b, we investigated whether the presence of difficult pseudocompounds, consisting of combinations of existing nouns, would tax the decomposition and subsequent reassembly of opaque compounds, thereby reducing the RT advantage over simple words. Note that we did not observe such a reduction for transparent compounds, in the comparison of Experiments 1a and 1b.

## Experiment 2b

### Method

#### Participants

The experiment was conducted with 31 native speakers of German (mean age = 25 years, 18 females) from the same population described above. They had normal or corrected-to-normal vision and participated for € 3.00 or course credit in our study.

#### Materials

Similar to Experiment 1b, 36 pseudocompounds made from real constituents (e.g., ^*^Schneemusik/^*^snowmusic) replaced the pseudocompounds used in Experiment 2a. This was the only change of the stimulus material in comparison to Experiment 2a. See Table 3 for examples of each stimulus class and an overview of stimulus properties, and Appendix for a list of the whole stimulus set.

#### Procedure

Time-out was set to 3000 ms to account for the different pseudoword type. No other aspects of the procedure were altered in comparison to Experiment 2a.

#### Results

Data handling was performed as before. Thirty-three compounds and 132 simple words out of 2232 existing words were discarded as “unknown.” The total error rate was 8.0%. See Table 4 for mean RTs and error rates of the different stimulus classes. The data of 27 participants and 29 item pairs entered further analysis. Planned *t*-tests did not detect any significant differences in mean RTs between compounds and morphologically simple words: all *t*s < 1. In an additional analysis, we combined the data from Experiments 2a and 2b, with Pseudoword Type (varied between experiments) as factor. The ANOVA showed an interaction between Word Type (opaque compound, monomorphemic word) and Pseudoword Type (easy, difficult): *F*_1(1, 57)_ = 5.844, *p* = 0.019, *partial* η^2^ = 0.093; *F*_2(1, 57)_ = 4.803, *p* = 0.033, *partial* η^2^ = 0.078.

#### Discussion

When the pseudowords consisted of non-existing combinations of two existing morphemes (e.g., ^*^snowmusic), semantically opaque compounds were processed only numerically faster than matched simple words, but the difference was not significant. This was corroborated by the interaction between word type (opaque compounds, simple words) and pseudoword type (easy, difficult), on the combined data from Experiments 2a and 2b. So, when decisions are easy, we observed a large effect of the constituent frequency of the opaque compounds, which was absent in the presence of difficult pseudocompounds. This pattern is different from what was observed for semantically transparent compounds. As with Experiments 1a and 1b, the presence of difficult pseudocompounds slowed down reactions considerably for compounds and monomorphemic words, but in contrast to Experiments 1a and 1b (transparent compounds), the increase was much larger for opaque compounds (161 ms) than for simple words (108 ms). Both pieces of evidence point to the same: When pseudocompounds are difficult to reject, RTs to existing words increase, and the constituent-frequency advantage for semantically opaque compounds is reduced to statistical insignificance. We will take up this point in the general discussion of our findings.

## General discussion and conclusions

Our experiments focused on the question whether visually presented morphologically complex words in German are parsed into their constituent morphemes during word recognition, and, if so, what costs occur as a consequence of decomposition. We also aimed to shed light on the possible influence of a complex word's semantic transparency with respect to these issues.

In many cases, lexical-decision latencies were faster for compounds than for matched monomorphemic words. This can be explained by fast and efficient access to the compounds' constituents, which were of higher frequency than the simple words, and the compounds themselves. This processing head start is found for semantically transparent and opaque compounds, which indicates that decomposition takes place well before semantics are at play. The presence of pseudowords such as “snow music” or “piano cup,” that prevent correct lexical decisions on the basis of the lexical status of the constituents alone, did not affect the advantage, over monomorphemic words, of transparent compounds with high-frequency constituents. But statistically it wiped out the head start for opaque compounds. First and foremost, the combined data from four experiments clearly support morphological parsing of compound words in German. None of the models that assume direct access to word-forms for all words—including morphologically complex words—can explain our results. Thus, our data do not support full-listing, as suggested by Butterworth (1983), nor the supra-lexical model put forward by Giraudo and Grainger (2000). In three experiments, we observed reliable differences between compounds and frequency- and length-matched monomorphemic words. Without sublexical decomposition, no advantage in RT should have been found for compounds, given that this advantage can only be due to access to the high-frequency constituents (see Andrews, 1986).

Our data fit well with models proposed by Taft (e.g., Taft, 2004; Taft and Ardasinski, 2006; Taft and Nguyen-Hoan, 2010), and with dual-route models (Caramazza et al., 1988; Schreuder and Baayen, 1995; Baayen and Schreuder, 1999), because these models allow for decomposition. Taft and colleagues claim that decomposition is an obligatory and automatic process during visual word recognition. Caramazza and colleagues, as well as Schreuder and Baayen, favor the idea of multiple pathways to word recognition—via direct access or via decomposition, with many parameters contributing to which route will be taken. In the most recent, multiple-route model (Kuperman et al., 2009), lexical access is achieved by an interactive process in which all (morphological) cues are used as soon as they become available. The parallel processing in this multiple-route model contrasts with the sequential approach of Taft and colleagues, and allows for access to semantic features without decomposition.

To disentangle obligatory decomposition and dual-route models, we designed our pseudowords from Experiments 1b and 2b in such a way that the task should be difficult to accomplish. These pseudowords were built from existing stems (e.g., piano cup, dream burden, written together in German). The logic for the use of such pseudowords is to force decomposition and reassembly (cf. Taft, 2004). Of course, such pseudowords could be looked up, and no full-form entry would be found. If this is what happens, they should be no more difficult to distinguish from real words than pseudowords made up from real words and non-words. In fact, pseudoword compounds made from existing words yielded longer latencies and higher error rates than pseudowords made from words and non-words. Taft and colleagues have shown that the use of pseudowords constructed from existing morphemes taxes the process of reassembly of the (parsed) morphemes of existing complex words, to the extent that decisions to such words actually take longer than to matched monomorphemic words (Taft, 2004). This is not what we found, but we do observe an overall slowing, and, importantly, the disappearance of what we called the head start for compounds over simple words. Even if this results in latencies that are very similar to those of matched monomorphemic words, which obviously invites an interpretation in terms of full-form access, we believe that our data rather show decomposition and subsequent costs of reassembly into a whole-word unit. One reason concerns the role of the different types of pseudowords just mentioned. Another reason concerns the very low full-form frequency of the compounds, and the much higher frequency of their constituents (even for the low-frequency compounds of Experiment 1), which would favor the decomposition path even in dual-route models.

Although the model proposed by Taft (Taft, 2004; Taft and Ardasinski, 2006) was not developed on the basis of data from compound processing, we believe that it perfectly explains our results. Taft reasons that the recombination stage—implemented at a lemma level—is crucial in explaining the processing costs occurring in visual word recognition. As in the two-stage model of language production (Levelt et al., 1999), the lemmas are whole-word units, integrating constituent morphemes, and providing access to semantic and functional (syntactic category, gender, and so on) information. If a complex word carries more or slightly different semantic information than its constituents, a lemma is needed over and above the lemmas for the constituents. We believe that these integrating units are needed for all compounds, because every compound, even a transparent one, has additional meaning over and above the constituents' meaning (e.g., *cup*, *cake*, and *cupcake*). In addition, only compound lemmas can code or point to the type of relation between the constituents (part of, made of, good for, and so on; Gagné and Spalding, 2009). For fully transparent compounds, the constituents' lemmas will activate their concepts which—being semantically related to the whole word's meaning—may facilitate word recognition for the compound. Differences in task difficulty mediate the processing costs at the recombination stage, but the head start gained from high-frequent constituents is not cancelled out by this.

If decomposed (and the data from Experiment 2 clearly speak in favor of decomposition), the meaning of semantically opaque compounds can only be accessed via an integrating unit—the compound's lemma, in Taft's model. The fact that the meaning of its constituents is also activated may well-hamper its recognition, because the constituents do not contribute to the opaque compound's meaning. This may result in longer latencies for opaque than for transparent compounds. Still, in an environment that does not overly tax the recombination stage (our “easy” pseudoword-task), the head start gained from high-frequent constituents impacts on RT, and effects of decomposition are clearly visible. If however, a lot of pressure is laid on the recombination stage by the presence of difficult pseudowords, and if semantic activation of the constituents rather hinders than helps recognition, the advantage of decomposition is consumed by higher integration costs, as can be seen in our Experiment 2b data. According to Taft (2004), latencies might even be longer for morphologically complex words than for monomorphemic words, depending on the demands at the recombination stage. Experimental environment is a factor that definitely influences the processing costs during the recombination of constituents.

Our results corroborate those reported by Fiorentino and Poeppel (2007), and support early morphological parsing. They are in line with the findings reported by Ji et al. (2011), who also found strong evidence for decomposition, and for processing costs depending on experimental environment and semantic transparency with English stimuli. Our data clearly corroborate these findings with data from a different language. They are also in line with the role of visual acuity in decomposition, reported by Bertram and Hyönä (2003) and Hyönä (2012), who found decomposition for long Finnish compounds, but not for short ones, depending on whether stimuli can be perceived within one fixation. On the other hand, there is also evidence for the existence of whole-word representations in visual word recognition. For example, Baayen et al. (1997) found evidence for whole-word form storage of Dutch plurals, stating that full-form storage might be better than time-consuming reintegration of constituents, and also Pollatsek et al. (2000) report data for Finnish that can be more easily handled by a dual-route model than by a model of obligatory decomposition.

Evidently, full forms exist for complex words, provided that they have been encountered often enough, and thus have a high surface frequency. This certainly does not apply to our compounds.

To sum up, our results provide strong evidence for decomposition in the processing of German compounds. This is evident from the positive reaction-time effects due to the high frequency of compound constituents. Our data also support the existence of a reassembly stage, at which the constituents are integrated into a unitary representation for the full compound that specifies the unique semantic, combinatorial (and syntactic) properties of compounds. When the reassembly process is heavily taxed, for example by the presence of pseudocompounds that consist of two existing stems, the processing advantage due to frequent constituents may be diminished or completely swallowed. The difference observed between semantically transparent and opaque compounds in a taxing experimental context may be explained by the positive contribution of the semantics of constituents to the meaning retrieval for the compound as a whole. The full pattern of results best fits with the model put forward by Taft and colleagues, developed for English derived words (e.g., Taft, 2004; Taft and Ardasinski, 2006; Taft and Nguyen-Hoan, 2010).

### Conflict of interest statement

The authors declare that the research was conducted in the absence of any commercial or financial relationships that could be construed as a potential conflict of interest.

## Appendix

### Materials

**Table:**

**1a and 1b Compounds “high”**	**1a and 1b Compounds “low”**	**1a Simple words**	**1b Simple words**	**2a and 2b Compounds “opaque”**	**2a and 2b Simple words**
Armbrust	Armbinde	Grimasse	Grimasse	Seekuh	Waffel
Baumhaus	Baumkrone	Anakonda	Anakonda	Tagebau	Harpune
Bettdecke	Bettlaken	Kannibale	Kannibale	Feldbett	Vaseline
Biermarke	Bierkrug	Trichter	Trichter	Mundraub	Dromedar
Feuergott	Feuerpfeil	Terpentin	Appalachen	Maulwurf	Schleuse
Handgriff	Handschuh	Trampolin	Trampolin	Lackaffe	Zwetsche
Heuhaufen	Heuballen	Therapeut	Therapeut	Luftloch	Liguster
Luftstrom	Luftpumpe	Mandarine	Mandarine	Sägemehl	Kastanie
Türrahmen	Türöffner	Akropolis	Akropolis	Angsthase	Aubergine
Wasserfall	Wasserhahn	Salamander	Salamander	Pagenkopf	Tolpatsch
Wortspiel	Wortwitz	Integral	Mysterium	Kammerton	Hagebutte
Seegang	Seeigel	Torpedo	Torpedo	Spaßvogel	Pestilenz
Fußform	Fußpfad	Geranie	Geranie	Kriegsfuß	Engerling
Hofdame	Hofnarr	Stummel	Stummel	Muttermal	Basilikum
Kuhhaut	Kuhmist	Kamille	Kamille	Stuhlgang	Therapeut
Ölaktie	Öllampe	Termite	Termite	Zaunkönig	Flittchen
Sandburg	Sanddüne	Speiche	Speiche	Kotflügel	Wacholder
Teezeit	Teesieb	Kompott	Luzerne	Löwenzahn	Harmonika
Feldrand	Feldhase	Harlekin	Harlekin	Sauwetter	Hortensie
Autodach	Autodieb	Orchidee	Orchidee	Bergkette	Scharnier
Bergwelt	Bergdorf	Marzipan	Schnecke	Baumschule	Scharlatan
Blutfluss	Blutegel	Hermelin	Hermelin	Geizkragen	Katakombe
Eiskanal	Eisdiele	Etikette	Etikette	Glückspilz	Schnorchel
Gasmarkt	Gaspedal	Geländer	Geländer	Augenweide	Terrakotta
Geldsumme	Gelddepot	Paradigma	Paradigma	Seifenoper	Frikadelle
Glasauge	Glassarg	Hibiskus	Hibiskus	Schlitzohr	Bergamotte
Laubfrosch	Laubsauger	Frikadelle	Frikadelle	Adamsapfel	Salamander
Mondmann	Mondflug	Kontinenz	Hagebutte	Donauwelle	Patisserie
Obststand	Obsternte	Scharlach	Scharlach	Gürtelrose	Kasserolle
Sojamilch	Sojabohne	Eidechse	Eidechse	Hosenboden	Alabaster
Pilzsuche	Pilzzucht	Wacholder	Wacholder	Schlagwort	Petersilie
Postfach	Postbote	Thermostat	Kassette	Sternstunde	Lapislazuli
Salzstock	Salzsäure	Alligator	Alligator	Silberblick	Schaschlik
Zaunkönig	Zaunpfahl	Barrikade	Barrikade	Trommelfell	Geschwulst
Tanzfilm	Tanzbar	Kolibri	Kolibri	Pustekuchen	Klavichord
Weinbau	Raubbau	Tablett	Tablett	Kaiserschnitt	Rhododendron
Landweg	Waldweg	Almosen	Almosen		
Kinokarte	Menükarte	Kakerlake	Kakerlake		
Tierhaar	Kamelhaar	Hortensie	Hortensie		
Segeltuch	Leintuch	Aprikose	Aprikose		
Banknote	Duftnote	Schnorchel	Holunder		
Festball	Federball	Bakterie	Bakterie		
Papierhut	Zauberhut	Margerite	Margerite		
Windrad	Zahnrad	Flieder	Flieder		
Startbahn	Seilbahn	Korridor	Korridor		
Halsband	Gummiband	Bratsche	Bratsche		
Tischbein	Steißbein	Aubergine	Aubergine		
Kursbuch	Notizbuch	Bumerang	Bumerang		
Brotdose	Puderdose	Alabaster	Alabaster		
Winterfell	Schafsfell	Artischocke	Palisander		
Zielfoto	Farbfoto	Mahagoni	Mahagoni		
Turmuhr	Atomuhr	Wachtel	Wachtel		
Zellgift	Nagergift	Languste	Languste		
Nachthemd	Polohemd	Schablone	Schablone		
Sporthose	Jeanshose	Kartusche	Kartusche		
Messejahr	Dürrejahr	Tolpatsch	Tolpatsch		
Schulkind	Patenkind	Schatulle	Schatulle		
Warenkorb	Strandkorb	Kautschuk	Kautschuk		
Staubkorn	Hirsekorn	Katakombe	Katakombe		
Datennetz	Gleisnetz	Pharmazie	Rhabarber		
Holzofen	Glutofen	Rapunzel	Rapunzel		
Elternpaar	Brautpaar	Marionette	Schimpanse		
Kunstpelz	Biberpelz	Zwetschge	Zwetschge		
Stadtplan	Terminplan	Klarinette	Klarinette		
Samtrock	Tüllrock	Begonie	Begonie		
Sachpreis	Taxipreis	Terrakotta	Terrakotta		
Metallrohr	Schilfrohr	Koordinate	Bergamotte		
Bordwand	Felswand	Schnalle	Schnalle		
Platzzahl	Wattzahl	Rhesus	Quotient		
Tatabend	Infoabend	Margarine	Margarine		
Rauchalarm	Ozonalarm	Kasserolle	Kasserolle		
Rosenblatt	Kleeblatt	Zentrifuge	Geschwader		
